# Melatonin Exerts Anti-Inflammatory, Antioxidant, and Neuromodulatory Effects That Could Potentially Be Useful in the Treatment of Vertigo

**DOI:** 10.1155/2021/6641055

**Published:** 2021-03-23

**Authors:** Joaquin Guerra, Jesus Devesa

**Affiliations:** ^1^Otolaryngology, Foltra Medical Center, Travesía Montouto 24, Teo 15886, Spain; ^2^Scientific Direction, Foltra Medical Center, Teo 15886, Spain

## Abstract

The acute phase of vertigo involves multiple neurotransmitters, inflammatory mediators, and products of oxidative stress. The vestibular pathway has multiple melatonin receptors distributed along its path, both centrally and peripherally. In addition, melatonin has been shown to be a powerful antioxidant and anti-inflammatory agent against factors related to vertigo, such as Bax/caspases, interleukins, and chemokines. Likewise, it exerts central GABAergic, antidopaminergic, and anti-migraine functions and regulates sympathetic activity in a similar way to the drugs classically used in acute vestibular crisis. In this review, the role of melatonin as a potential treatment of the acute phase of vertigo is discussed.

## 1. Introduction

The acute phase of vertigo appears in several vestibular syndromes with different pathophysiology, such as Menière's disease (MD), vestibular neuritis (VN), vestibular migraine (VM), and benign paroxysmal positional vertigo (BPPV). Due to the involvement of the inner ear, the symptoms may include not only recurrent attacks of vertigo but also fluctuating hearing loss or tinnitus. In addition, multiple central nervous system conditions associated with migraine or cerebrovascular and neurodegenerative disorders have been described, which can manifest vertiginous attacks [1, 2].

Vestibular syndromes imply that metabolic factors may act in their development, such as (1) multiple neurotransmitters with different effects: excitatory (glutamate, dopamine, and serotonin), modulating (histamine and enkephalins), or inhibitory (GABA and glycine); (2) inflammatory cytokines (TNF and IL3); (3) reactive oxygen species (ROS); and (4) other factors [3–5].

Although the different types of vertigo imply a different pathophysiology, the treatment of the acute crisis is usually symptomatic, which implies a similar pharmacological management, which generally acts by exerting a sedative effect. For this reason, among others, antihistamines, anticholinergics, benzodiazepines, or antidopaminergic drugs are used [3]. However, considering that the incidence of vertiginous syndrome is particularly higher in the elderly population, in this and other risk groups, the use of a lower dose of the aforementioned drugs or the use of other treatments with fewer side effects could be useful [6].

That is why in this study, we will analyze the possible role that melatonin, a harmless hormone, could play in regulating the acute phase of vertigo.

## 2. Melatonin and the Vestibular Pathway

Melatonin performs extensive functions not only in the inner ear but also in the vestibular pathway, regulating its function. Precisely, in the inner ear, there is a wide and diffuse expression of melatonin MT1 receptors, distributed in many structures, including the organ of Corti, the spiral and vestibular ganglion, vestibular sensory cells, dark vestibular cells, transitional cells, or epithelial cells of the endolymphatic sac [7]. With respect to the central vestibular structures, MT1 and MT2 receptors are also found in the vestibular nuclei, the thalamic vestibular pathway, and the cerebral and cerebellar cortex [8]. The cerebellum expresses the highest number of melatoninergic receptors [9]. Clinically, melatonin receptors found in the area postrema, a structure in the medulla oblongata of the brainstem, can modulate vomiting and other sympathetic responses that characterize the attack of vertigo [10]. The paraventricular nuclei and the reuniens connect with the limbic system, and this may be the reason for their regulation in mood and sedation, relevant elements for the control of vertigo [11].

Furthermore, several reports have attempted to explain the disorganization of circadian rhythms in patients with vestibular disorders. For example, aging implies a degeneration of the vestibular pathway, in which the dysregulation of the circadian rhythm could influence the genesis of vestibular imbalance [12]. Furthermore, melatonin has been proposed as a prophylactic agent in the prevention of migraine attacks, a condition that can be associated with vertigo [1, 2, 13]. In addition, in patients with bilateral vestibular loss, there is a lack of synchronization between temperature and the rest-activity cycle, which affects the physiology of melatonin regulation [14]. Although the effect of melatonin can be exerted by direct action, it is true that it has the potential to modulate other compounds, enhancing or inhibiting them, and thus their actions [15, 16].

## 3. Role of Melatonin as an Anti-Inflammatory and Antioxidant Vestibular Agent

Vertigo patients show higher levels of reactive oxygen species (ROS) and superoxide metabolites than healthy subjects, as shown by multiple reports from subjects with different vestibular syndromes, such as BPPV, MD, or unspecified situations of chronic subjective vertigo. It includes higher levels of hydrogen peroxide, oxidation products of thiol and other ROS, and lower activity of superoxide dismutase (SOD), glutathione content, and catalase [17–21]. Oxidative stress may be due to the physiological stress that vertigo induces [18]. The antioxidant effect of melatonin is well known, since it acts as a direct scavenger of free radicals with the ability to detoxify both reactive oxygen and reactive nitrogen species [22]. In animal models, the otoprotective function of melatonin has been demonstrated after exposure to gentamicin in the inner ear; this otoprotection is mainly based on the inhibition of the genesis of free radicals or scavenging them. Gentamicin induces an increase in the levels of ROS and proapoptotic Bcl-2-associated protein *X* (Bax) in utricular hair cells, in turn inhibiting the expression of B-cell lymphoma 2 (Bcl-2). Melatonin reverses this event by inhibiting the expression of caspase-3. This protein is essential in the activation of programmed cell death [23].

Interestingly, in patients with chronic subjective dizziness, an inflammatory response with elevated serum levels of tumor necrosis factor *α* (TNF) and interferon *γ* (IFN*γ*) has been reported [20]. Along with similar lines, patients with MD show an elevation of various interleukins (IL-1*β*, IL-1RA, and IL-6) and TNF baseline levels. Furthermore, in these patients, the two subgroups can be differentiated according to their IL-1*β* profile; those with higher basal levels exhibit increased levels of cytokines and chemokines (CCLs). Interestingly, the proinflammatory immune response appears to increase in those subjects exposed to allergenic extracts of Aspergillus and Penicillium involving TNF, which points to a possible allergic association [24].

According to these reports, the levels of IL-1*β*, CCL3, CCL22, and CXCL1 have been proposed as differentiating markers of MD from other vestibular syndromes that can confuse the diagnosis, such as VM, whose clinical expression can be very similar [25]. In VN, the CD40 receptor, which belongs to the family of TNF, and its ligand (CD40L) have been suggested to be involved in the progression and genesis of the disease, thus increasing the production of several proinflammatory cytokines, such as TNF [26]. As described above, vestibular syndromes exhibit inflammatory reactions during acute attacks and subjects with chronic vertigo have higher basal levels of inflammatory mediators, so that melatonin theoretically would be able to regulate not only attacks but also recurrences, given its regulation of the release of various cytokines. Although no report has specifically focused on the role of melatonin in the vestibular system and these cytokines, this hormone could centrally or peripherally control the levels of CCLs, ILs, and TNF [27]. For example, while central IL-1*β* suppresses the physiological nocturnal secretion of melatonin [28], exogenous administration of this hormone reduces the inflammatory activity of IL-1*β* [29] by upregulating the expression of superoxide dismutase (SOD) and downregulating the expression of Bax [30]. Inhibition of IL-1*β* and TNF has also been reported to downregulate PI3K/AKT, ERK, NF-*κ*B signaling pathways, as well as overexpression of miR-3150a-3p [31]. Other studies show that melatonin inhibits the gene expression of other cytokines or their receptors, such as IL-1RA, or the expression of CCL induced by LPS in different inflammatory conditions [32, 33].

**Figure 1:**
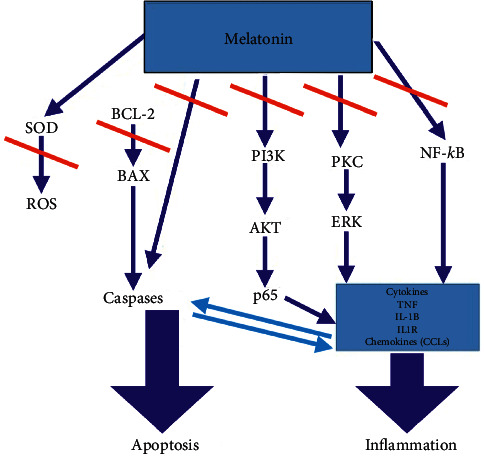
Potential anti-inflammatory and antioxidant actions of melatonin in vestibular disorders. Blue arrows indicate stimulation or activation of some enzymes, signaling pathways, inflammation markers, and genes, while the red arrows indicate the inhibitory effects of melatonin on these pathways, thus inhibiting apoptosis and inflammation. Note how melatonin activates SOD.

As shown in Figure 1, melatonin may exert beneficial effects by blocking the activity of vestibular oxidative and inflammatory stress through several pathways.

## 4. Melatonin as a Modulator in the Vestibular Neurotransmission

Gamma-aminobutyric acid (GABA) is the predominant inhibitory neurotransmitter in the vestibular pathway. Of the three GABA receptors described, GABA-A and GABA-B are involved in vestibular neurotransmission [34]. Studies show that GABA plays a plausible role in inner ear afferent transmission, but its role as the primary transmitter at this level is unclear. It is accepted that its function is to modulate neuronal transmission, through the presynaptic inhibition of Ca^2+^ channels and/or the activation of Cl channels. Therefore, it can indirectly decrease the release of presynaptic neurotransmitters to affect the excitability of postsynaptic cells [35, 36]. The central vestibular nuclei receive inhibitory inputs that are mediated by GABA-A and GABA-B receptors. These GABA-A inputs arise primarily from the commissural fibers of the vestibular nuclei and the cerebellum [37]. Theoretically, the treatment with agonists of the GABA-A (benzodiazepines) and GABA-B (baclofen) receptors is based on an effect on the central vestibular sensory pathways [38]. Melatonin can also regulate the GABAergic synaptic transmission and thus modulates the activity of its receptor [39]. Its sedative effect is mainly enabled by binding to the GABA-A receptor, as it occurs with benzodiazepines [40]. This sedative action may induce a decrease in blood pressure [41]. In rats submitted to pinealectomy, the interruption of the cerebral circadian rhythm of this neurotransmitter prevents the union of benzodiazepines to their receptors; with the use of exogenous melatonin, this phenomenon is reversed [42]. In vitro studies have also shown that melatonin increases the amplitude and frequency of miniature postsynaptic current inhibitory chemicals (mIPSCs), enhancing the hippocampal GABAergic transmission [43].

Several findings support a possible involvement of dopamine as a modulator of excitatory vestibular neurotransmission in the postsynaptic afferent terminals in at least 2 of the 5 dopamine receptors identified. In the vestibular neuroepithelium of mammals, immunochemical tests show that D1 and D2 receptors (coupled to *G* proteins) are expressed in the vestibular hair cell membranes [44]. The responses of these receptors not only modulate postsynaptic glutamate receptors but may also have a protective function on vestibular dendrites [45]. The existence of dopamine D2 receptors has been reported in the vestibular nuclei [46]. The use of antidopaminergic drugs (sulpiride and prochlorperazine) exerts a modulating effect on vestibular neurons and controls vomit [3, 47]. Although there is no report directly involving melatonin in this effect in vestibular structures, it has been demonstrated that this hormone modulates dopamine and can inhibit its release in specific areas in the CNS of mammals, such as the hypothalamus, hippocampus, striatum, medulla-pons, and retina [48].

Other compounds involved in vestibular neurochemistry, such as substance P or calcitonin gene-related peptide (CGRP), both implicated in migraine, and thus potentially vestibular migraine (VM), are also inhibited by melatonin [49–51]. Furthermore, TNF stimulates CGRP transcription, whereas as previously described, melatonin is capable of inhibiting TNF release [31, 50]. CGRP antagonists are currently being developed for the treatment of migraine, although they should not be considered as first-line treatments [52].

Table 1 summarizes the potential effects of melatonin on neurotransmitters presumably involved in the genesis of vertigo.

## 5. Regulation of Melatonin in the Vestibular Sympathetic Activity

Melatonin release is controlled by the sympathetic innervation of the pineal gland, which mediates the inhibitory effect of light on pineal melatonin secretion. This pathway begins in the retina, influencing the biological clock of the suprachiasmatic nucleus [53], and then inhibits the paraventricular nucleus and interrupts the stimulation of the intermediolateral nucleus, inducing melatonin synthesis [54]. As previously described, the physiology of melatonin is closely related to the sympathetic nervous system and sympathetic activity correlates with circadian rhythms [55]. For example, the cardiovascular response to laryngoscopy and laryngotracheal intubation is attenuated by the administration of melatonin [41].

Patients diagnosed with vertigo show less parasympathetic activity; the ratio of sympathetic/parasympathetic activity is higher than in healthy subjects [18]. The effects of melatonin on the autonomic system cause a reduction in the adrenergic flow [56] and induce relaxation of the smooth muscle of the arterial wall by increasing the availability of nitric oxide [57]. Furthermore, melatonin is capable of lowering blood pressure, specifically binding to its MT1 and MT2 receptors in blood vessels, thus blocking the catecholaminergic response [58].

Pinealectomized rats show higher circulating levels of catecholamines when stimulated with IL1-*β*, an inflammatory mediator involved in vertigo, as mentioned previously [24]. This effect is reverted after intraventricular infusion of melatonin [59]. In spontaneously hypertensive rats, acute administration of melatonin lowered blood pressure along with norepinephrine levels [60], although the authors found that this effect might not be mediated by melatonin receptors or *α*-adrenoceptors, but by the antioxidant effect of melatonin that inhibits inositol phosphate [61]. In humans, exogenous use of melatonin has been shown to be effective in reducing circulating catecholamine levels [62], as well as blood pressure, carotid pulsatility index [63], and sympathetic nerve responses to orthostatic stress [64].

## 6. Conclusions

Based on the data included in this review, it seems obvious that the use of melatonin in the acute phase of vertigo can be highly effective, although more studies and clinical trials are needed. However, despite the fact that the effect in humans may be more limited than in laboratory animals, it is evident that the adjuvant use of melatonin with other drugs could not only improve the vestibular symptoms of acute vertigo crisis but also prevent the increase of doses of commonly used drugs with the consequent increase in pharmacological toxicity. This type of combined treatment would be especially indicated in risk groups, such as the elderly population. Moreover, melatonin is a practically harmless hormone; the lethal dose 50 could not be found yet. Paradoxically, some reports showed transient dizziness as a side effect. This symptom may be only a subjective report or it may be associated with its sedative function, and it does not limit its use as with other drugs employed in acute vertigo [65]. However, we cannot ignore the possibility of undesirable effects appearing in patients who are recovering after an attack of acute vertigo, delaying vestibular compensation. These adverse effects have been observed in posturographic results and in oculomotor tests, with a decrease in saccade accuracy or smooth pursuit gain [66]. Furthermore, the decreased sympathetic response may theoretically exert a greater intolerance to orthostatism, although this conclusion may be questionable [64]. Moreover, there are no reports of vestibular worsening demonstrated in neurophysiological tests, such as vestibular evoked myogenic potential (VEMP) [67]. As previously described, its safety profile, even at extremely high doses, is wide [65].

Although systemic administration is safe and favors effects on different organs of the vestibular pathway, it remains to be seen whether topical (transtympanic) administration could be effective for pathologies of peripheral origin. A route of entry for various metabolites with oxidizing or inflammatory power is the round window. The main advantage of this approach relies on the fact that melatonin would perfuse directly to the inner ear, as it occurs with the intratympanic corticosteroid treatment. Moreover, treating melatonin topically could minimize the effect of mediators that access through this route of entry, implied in the development of vestibular syndromes such as labyrinthitis or endolymphatic hydrops [68]. In conclusion melatonin administration in vertigo could be a new therapeutic effect of melatonin, among the many already described that this hormone exerts in human pathologies.

## Figures and Tables

**Table 1:** Melatonin effects on neurotransmitters.

Neurotransmitter	Vestibular receptors	Melatonin action	Effect
GABA	GABA-A	Stimulation	Sedative
	GABA-B		Anxiolytic

Dopamine	D1	Inhibition	Sedative
	D2		Antiemetic

Substance P	NK1R	Inhibition	Anti-inflammatory
			Anti-migraine

CGRP	CALCRL	Inhibition	Anti-inflammatory
			Anti-migraine

## Data Availability

No data are used to support this study.
